# A Novel Mutation in the NAGLU (N-Acetyl-Alpha-Glucosaminidase) Gene Associated With Mucopolysaccharidosis Type III-B in a Saudi Girl

**DOI:** 10.7759/cureus.30519

**Published:** 2022-10-20

**Authors:** Rawia F Albar, Rahaf A AlQurashi, Nada Naaman, Atheer Alghamdi, Sara K Alghamdi, Khulud Aljohani, Rawaf Alsharif

**Affiliations:** 1 Pediatrics, King Abdulaziz Medical City, Jeddah, SAU; 2 College of Medicine, King Saud bin Abdulaziz University for Health Sciences, King Abdullah International Medical Research Center, Jeddah, SAU

**Keywords:** mucopolysaccharidosis type lllb, mps lllb, mps lll, autosomal recessive disease, sanfilippo syndrome

## Abstract

Mucopolysaccharidosis type III-B (MPS III), also known as Sanfilippo syndrome, is a rare autosomal recessive lysosomal storage disease that primarily affects the brain and spinal cord. In this report, we describe the case of an eight-year-old female child who presented to the emergency room with an asthma exacerbation. She had coarse facial features, thick eyebrows, deep-seated eyes, thinning coarse hair, and macrocephaly. Moreover, she suffered from hepatosplenomegaly, generalized muscular atrophy, global developmental delay, and scoliosis. Urinary glycosaminoglycans (GAGs) were within normal limits. Full genetic testing confirmed the diagnosis of Sanfilippo syndrome type B with a deficiency of alpha-N-acetylglucosaminidase caused by a homozygous mutation c.889C>T, p.(Arg297*) in the NAGLU (N-acetyl-alpha-glucosaminidase) gene. Chromosomal microarray analysis (CMA) showed a copy number variant (CNV) within the 1q24 region. Thus far, CNVs similar in size and position have not been reported in the literature, making this a novel mutation.

## Introduction

Mucopolysaccharidoses (MPS) are lysosomal storage diseases characterized by the deficiency of the enzymes required for the degradation of mucopolysaccharides, which are also known as glycosaminoglycans (GAGs) [1]. MPS is classified into several types based on the specific lysosomal enzyme affected. The different MPS subtypes show variable phenotypes and severity [2]. MPS III, also known as Sanfilippo syndrome, is a rare condition that leads to multisystem deterioration including childhood-onset neurodegeneration, organ dysfunction, skeletal deformities, and a usual life expectancy lasting up to the second or third decade of life [3]. The disorder is inherited in an autosomal recessive pattern and is characterized by the accumulation of the mucopolysaccharide, heparan sulfate [3]. MPS III is further subdivided into four subtypes based on the deficient lysosomal enzyme involved. The four enzymes are heparan N-sulfatase, alpha-N-acetylglucosaminidase, acetyl CoA:alpha-glucosaminide acetyltransferase, and N-acetylglucosamine-6-sulfatase [2]. The accumulated heparan sulfate in lysosomes leads to the clinical picture of the disease [1]. A systematic review reported that the lifetime risk at birth for MPS III ranges from 0.17 to 2.35 per 100,000 live births, and in Saudi Arabia, the risk at birth was estimated to be around 2 per 100,000 live births [4]. In this case report, we describe the manifestations of Sanfilippo syndrome type B in an eight-year-old girl of Saudi origin.

## Case presentation

An eight-year-old female child presented to the emergency department with complaints of cough and fever, with a clinical examination consistent with asthma exacerbation. The child was born to consanguineous Saudi parents after a full-term pregnancy and delivered by spontaneous vaginal delivery. The child was born to a 43-year-old mother whose pregnancy was complicated by oligohydramnios at 16 weeks of gestation.

**Figure 1:**
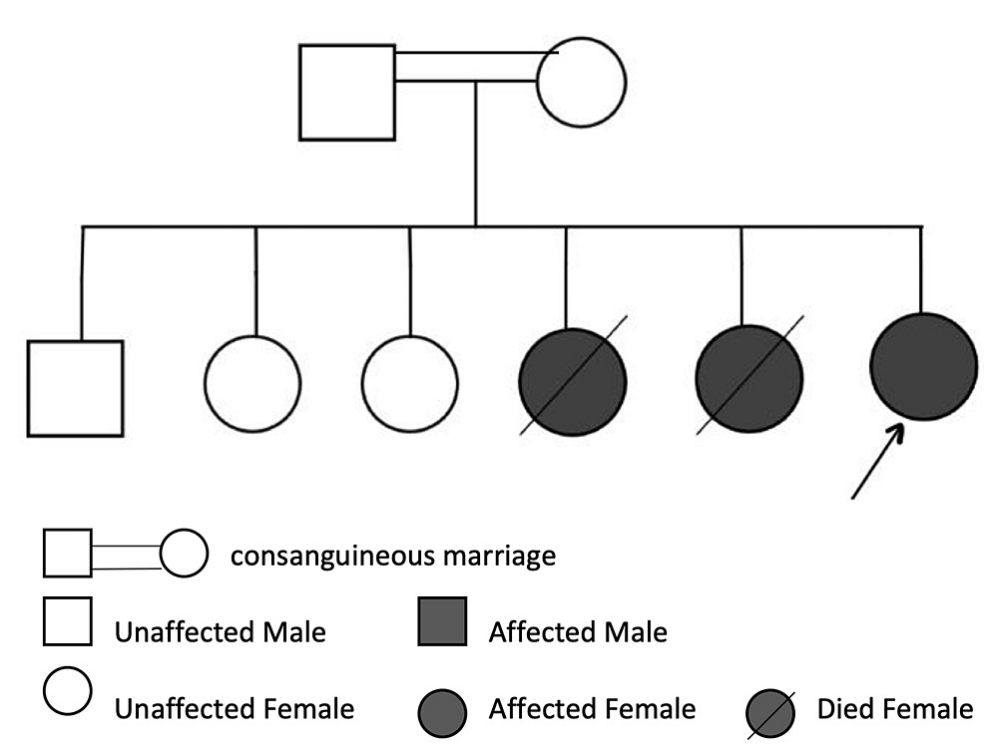
Family pedigree. Arrow indicating our case.

The child had intrauterine growth restriction with birth weight (1.6 kg), a delayed first cry, and was admitted to the neonatal intensive care unit and required mechanical ventilation for one month due to respiratory distress syndrome. The patient has three healthy older siblings (one brother and two sisters) and previously had two other sisters who were diagnosed with the same syndrome and died at the ages of 17 and 14 because of bleeding and infection, respectively. The family pedigree is shown in Figure 1.

The patient suffered from multiple comorbid conditions, including congenital cataracts, sensorineural hearing loss, cardiac defects (moderate patent ductus arteriosus, mild pulmonary hypertension, moderate tricuspid regurgitation, mitral stenosis, cardiomegaly, dilated left atrium and ventricle, and subaortic membrane with mild gradient), spasticity, subtle scoliosis, bilateral multicystic kidneys, atopy (bronchial asthma, eczema, and food allergies), and recurrent respiratory tract infections. According to the parents, she has had a global developmental delay with a delay in milestones (started walking at four years of age) and has had a general cognitive decline since birth. Her clinical condition deteriorated over time with oropharyngeal dysphagia and immobility, with a need for assistance for all activities. She had never been to school. The mother denied any aggressive behavior, autism, hyperactivity, sleep disturbance, or seizures.

**Figure 2:**
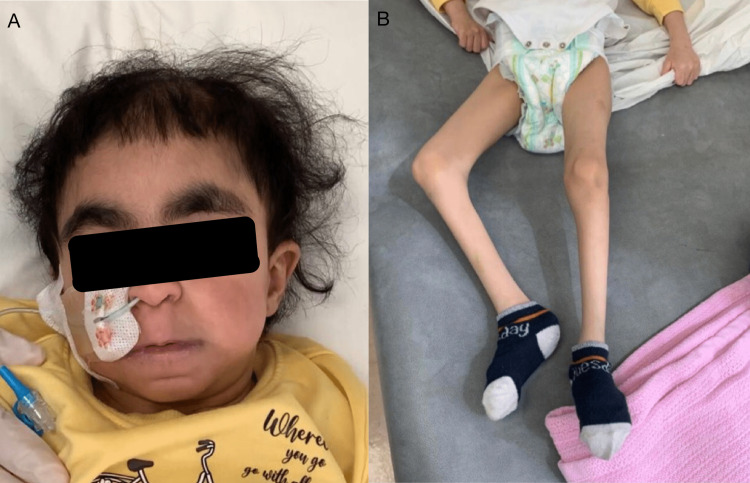
(A) Coarse facial features and (B) generalized muscular atrophy.

**Figure 3:**
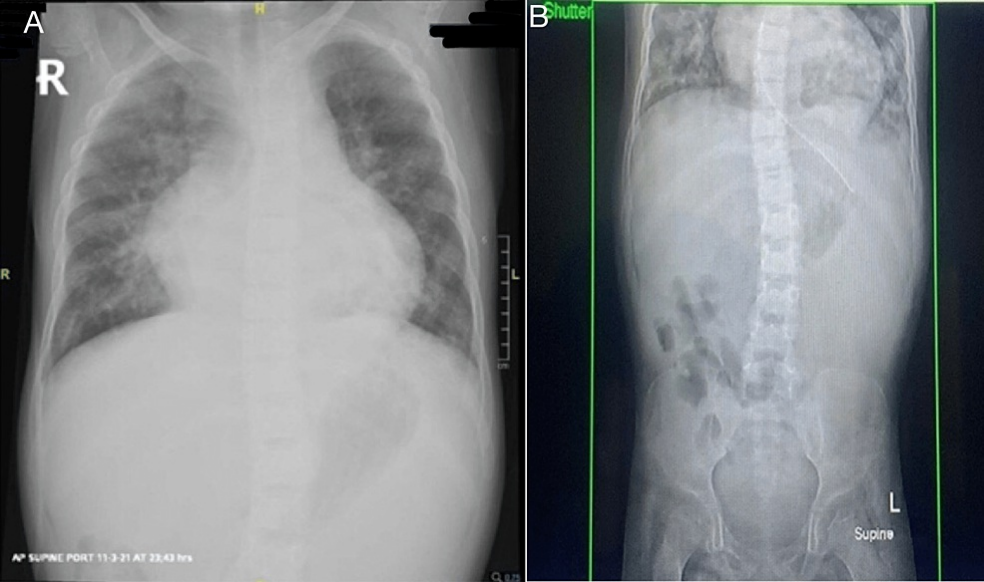
(A) Chest X-ray showing cardiomegaly and (B) abdominal X-ray showing scoliosis.

On examination, her weight was 10.6 kg, and her height was 92 cm, and she was wasted and stunted (BMI = 12.52, below the third percentile). She had coarse facial features, thick eyebrows, deep-seated eyes, thinning coarse hair, and macrocephaly (Figure 2). She had hepatosplenomegaly, generalized muscular atrophy, bilateral flexion contractures of elbows and knees, and scoliosis with fine tremors. Skeletal survey radiography showed diffuse osteopenia and increased trabeculation of the bone. A general laboratory workup revealed anemia and high creatinine levels, most likely due to undernutrition and muscle wasting. The rest of her labs were within normal limits. Radiography of the chest showed cardiomegaly with increased pulmonary vascularity (Figure 3).

The patient was diagnosed with Sanfilippo lllB at the age of 4 years, urinary GAG test was done for screening purposes. The result of the urinary GAG test was within normal limits. The patient was referred to a geneticist and a whole-exome sequencing (CentoXome Gold ®) uncovered a homozygous mutation c.889C>T, p.(Arg297*) in the NAGLU (N-acetyl-alpha-glucosaminidase) gene confirming the diagnosis of mucopolysaccharidosis type IIIB. This variant has previously been described as disease-causing for Sanfilippo syndrome B by Zhao et al., Yogalingam et al., and de Ruijter et al. [5-7].

Chromosomal microarray analysis (CMA; CentoArrayCyto™-HD incl. SNP test) was performed showing a copy number variant (CNV) within the 1q24 region. So far, CNVs similar in size and position have not been reported in the literature, therefore, making it a novel mutation. 

## Discussion

Only two cases of Sanfilippo syndrome have been reported in Saudi Arabia (types A and D) [8-9]. To the best of our knowledge, this is the first report of Sanfilippo syndrome type B from Saudi Arabia. In Table 1, we provided a summary of the clinical features previously reported with cases of Sanfilippo syndrome type B syndrome [10-17]. Most of the findings described in our patient were similar to those published in previous case reports. These clinical features include motor and speech development delays; facial dysmorphism, recurrent upper respiratory tract infections, hepatosplenomegaly, and cardiac defects. Besides the presented case, most cases reported in the literature were born to consanguineous parents [11,13,17], highlighting the strong association between consanguineous marriages and autosomal recessive disorders like Sanfilippo syndrome. Patients for whom brain magnetic resonance imaging (MRI) was performed showed similar findings of generalized volume loss. An exception was the study by Rezayi et al. [13], which presented primarily a picture of neurological deficits and a normal brain MRI. In the study by Rezayi et al. [13], the diagnosis of MPS was confirmed by the levels of GAG in the urine.

**Table 1:** Features of Sanfilippo type B as described in the literature and in our case. URTIs: upper respiratory tract infections, GAGs: glycosaminoglycans.

	Baldini et al. [10]	Elmas et al. [11]	Irigonhê et al. [12]	Rezayi et al. [13]	Hettiarachchi et al. [14]	Champion et al. [15]	Aydin et al. [16]	Federico et al. [17]	Our case
Consanguinity	-	+	-	+	+	+	+	+	+
Pregnancy and delivery complications	-	-	-	Not reported	-	-	Not reported	-	+
First signs and symptoms	Speech delay and behavioral problems	Speech delay, hepatomegaly, learning disability, ptosis of the left eye, and otitis media with effusion	Speech delay and loss of balance	Speech delay, seizures, and ataxia	Failure to thrive and developmental regression	Behavioral and sleep problems	Speech delay, vomiting, and restlessness	Progressive mental regression and hernia	Speech delay and vomiting
Age at diagnosis	Not reported	9 years	14 years		Not reported	4 years	3 years	Not reported	4 years
Behavioral problems	+	-	+	+	Not reported	+	+	Not reported	-
Sleep disorders	-	Not reported	+	Not reported	Not reported	+	Not reported	Not reported	-
Epilepsy	-	+	-	+	Not reported	Not reported	Not reported	Not reported	-
Hearing problems	Not reported	+	Not reported	Not reported	Not reported	+	Not reported	Not reported	+
Visual problems	Not reported	Not reported	Not reported	Not reported	Not reported	Not reported	Not reported	Not reported	+
Dysphagia	Not reported	Not reported	+	Not reported	Not reported	Not reported	Not reported	Not reported	+
Loss of sphincter control	Not reported	Not reported	+	Not reported	Not reported	Not reported	Not reported	-	+
Joint contractures	-	-	+	Not reported	Not reported	+	-	Not reported	+
Dysmorphic features	+	+	+	-	+	+	+	+	+
Skeletal abnormalities	Not reported	-	+	Not reported	Not reported	+	-	+	+
Cardiac anomalies	Not reported	+	+	Not reported	+	+	-	-	+
Renal anomalies	Not reported	Not reported	Not reported	-	Not reported	Not reported	Not reported	Not reported	+
Hepatosplenomegaly	+	+	+	Not reported	Not reported	+	+	+	+
Atopy	Not reported	Not reported	Not reported	Not reported	Not reported	Not reported	Not reported	Not reported	+
Recurrent URTIs	Not reported	+	+	Not reported	+	+	Not reported	+	+
Urine GAGs test results	-	+	+	+	Not reported	+	Not reported	+	-
Brain MRI findings	Not reported	Thinning of the corpus callosum	Ventriculomegaly, volume loss, and diffuse thickening of the diploe	Normal		Diffuse cortical atrophy on brain CT	Ventriculomegaly and volume loss	Ventriculomegaly on brain CT	Unavailable

One key difference present in our case was the negative urinary GAG test result. All reviewed reports had a positive test, except for the report by Baldini et al., in which the test was ordered when the patient was four years of age [10]. This suggests that a negative urinary GAG test does not rule out the diagnosis, especially when the test is ordered at a young age. Some differences between the cases may be attributed to the progressive nature of the disease. With the accumulation of substances like heparan sulfate, more symptoms may arise with increasing severity.

The variability in investigation results and patients’ presentations could cause a delay in the diagnosis of MPS III. In addition, patients could be misdiagnosed with unspecified metabolic disorders, autism spectrum disorders, epileptic disorders, or global developmental delays. Therefore, besides the clinical presentation, hospitals should utilize different investigation modalities such as laboratory data, genetic testing, and imaging findings to reach a proper diagnosis in a timely manner.

## Conclusions

Sanfilippo syndrome is a rare, progressive, genetic, multisystem, and lysosomal storage disease. While the pathophysiology is still not fully understood, it manifests as a result of the accumulation of glycosaminoglycans in body tissues and cells. Hence, patients typically present with features of developmental delay, speech delay, and intellectual disability. In addition, if clinical and laboratory findings suggest Sanfilippo syndrome in a child, further genetic and molecular testing must be conducted to confirm the diagnosis. In our report, we demonstrate the history and the important clinical findings of Sanfilippo syndrome type lllB in an eight-year-old girl at King Abdulaziz Medical City-Jeddah, Saudi Arabia. This case report is the first to describe Sanfilippo syndrome type B in Saudi Arabia.
